# Prediction models for chronic postsurgical pain in patients with breast cancer based on machine learning approaches

**DOI:** 10.3389/fonc.2023.1096468

**Published:** 2023-02-27

**Authors:** Chen Sun, Mohan Li, Ling Lan, Lijian Pei, Yuelun Zhang, Gang Tan, Zhiyong Zhang, Yuguang Huang

**Affiliations:** ^1^ Department of Anesthesiology, Peking Union Medical College Hospital, Chinese Academy of Medical Sciences & Peking Union Medical College, Beijing, China; ^2^ Outcomes Research Consortium, Cleveland, OH, United States; ^3^ Medical Research Center, Peking Union Medical College Hospital, Chinese Academy of Medical Sciences & Peking Union Medical College, Beijing, China

**Keywords:** chronic postsurgical pain (CPSP), breast cancer, prediction model, machine learning, high-risk identification

## Abstract

**Purpose:**

This study aimed to develop prediction models for chronic postsurgical pain (CPSP) after breast cancer surgery using machine learning approaches and evaluate their performance.

**Methods:**

The study was a secondary analysis based on a high-quality dataset from a randomized controlled trial (NCT00418457), including patients with primary breast cancer undergoing mastectomy. The primary outcome was CPSP at 12 months after surgery, defined as modified Brief Pain Inventory > 0. The dataset was randomly split into a training dataset (90%) and a testing dataset (10%). Variables were selected using recursive feature elimination combined with clinical experience, and potential predictors were then incorporated into three machine learning models, including random forest, gradient boosting decision tree and extreme gradient boosting models for outcome prediction, as well as logistic regression. The performances of these four models were tested and compared.

**Results:**

1152 patients were finally included, of which 22.1% developed CPSP at 12 months after breast cancer surgery. The 6 leading predictors were higher numerical rating scale within 2 days after surgery, post-menopausal status, urban medical insurance, history of at least one operation, under fentanyl with sevoflurane general anesthesia, and received axillary lymph node dissection. Compared with the multivariable logistic regression model, machine learning models showed better specificity, positive likelihood ratio and positive predictive value, helping to identify high-risk patients more accurately and create opportunities for early clinical intervention.

**Conclusions:**

Our study developed prediction models for CPSP after breast cancer surgery based on machine learning approaches, which may help to identify high-risk patients and improve patients’ management after breast cancer.

## Introduction

Breast cancer is the most common cancer in women. Although the ten-year survival rate of breast cancer has reached 82% (1, 2), there are still 20% to 60% of surviving patients experiencing chronic postsurgical pain (CPSP) after breast cancer surgery, resulting in a reduced quality of life and functional impairments (3–5). Predicting the risk of CPSP after breast cancer surgery can help clinicians identify those with a higher risk of CPSP and leading to earlier therapeutic interventions. In addition, identifying patients with a lower risk of CPSP could also prevent unnecessary therapy, saving limited medical resources.

Numerous factors have been found to be associated with CPSP after breast cancer surgery in the past decade, including social-demographic, intraoperative, and postoperative factors (6–8). Several models to predict CPSP have also been developed, mostly in European breast cancer patients (9–12). However, despite the acceptable discrimination and calibration of those models, the low clinical utility, especially positive predictive values (PPVs) around 0.2 at a 20~60% risk level of CPSP, limits their application in clinical practice, making it still difficult to identify high-risk patients early (9–12). Moreover, considering the different genetic, cultural and social backgrounds between different ethnic groups, which may also play an important role in this complex pathophysiological disease status, the extrapolation of those tools in Asian patients may be potentially limited.

Machine learning is a form of artificial intelligence that uses computer algorithms to identify nonlinear data patterns within large datasets to formulate outcome prediction and indicate improved prediction performance compared to the traditional prediction methods, which may be more suitable for the prediction of CPSP (13, 14). For example, random forest (RF) designs meta estimators that fit a number of decision tree classifiers on various sub-samples of the dataset and uses average to improve the predictive accuracy and control over-fitting. Gradient boosting decision tree (GBDT) gives a prediction model in the form of an ensemble of weak prediction models, which has strong generalization ability and performed well in both classification and regression tasks. Extreme gradient boosting (XGBoost) is an algorithm built on the GBDT framework and processed the missing data efficiently and flexibly (15). These three algorithms were also deep learning algorithms, the architecture of machine learning designed to mimic the neurological structure of the human brain, which might be more powerful than traditional algorithms in data analysis and prediction (16). Therefore, in this study, we intend to select these three algorithms with good fitting ability to develop prediction models for CPSP in Asian patients with breast cancer, and compare these models with logistic regression, hoping to improve the performance and clinical utility of the prediction models.

Contributions of this study:

1 This time we use machine learning approaches to develop prediction models, intending to improve the clinical utility and help clinicians identify high-risk CPSP patients more accurately and confidently.

2 Considering the different genetic, cultural and social backgrounds between different ethnic groups, which may also play an important role in CPSP, we focused on Chinese patients to explore predictors and models more suitable for Chinese population.

## Methods

### Study population

The study was a second analysis based on the dataset from the Chinese center in a multicenter randomized controlled trial (RCT, NCT00418457), held from 2014 to 2016, which has previously been described in detail (17–19). We enrolled women younger than 85 years with primary breast cancer without known extension beyond the breast and axillary nodes (ie, believed to be tumor stage 1-3, nodes 0-2) who were scheduled either for unilateral or bilateral mastectomy, with or without implants, or for wide local excision with node dissection. We excluded women who had previous surgery for breast cancer (we allowed diagnostic biopsies and guide-wire insertion), had inflammatory breast cancer, were scheduled for free-flap reconstruction, had American Society of Anesthesiologists (ASA) physical status of IV or higher, had contraindications to either anesthetic approach, or had other cancer not in long-term remission.

All the surgeries were conducted by the same surgical team, and the perioperative analgesia was standardized. In the original trial, patients were randomly assigned to either opioid analgesia (under fentanyl with sevoflurane general anesthesia, GA group) or paravertebral blocks (under paravertebral block with propofol general anesthesia, PPA group). Tramadol was the first-line postoperative analgesic in both study groups. Analgesia at home during the first postoperative week consisted of ibuprofen, acetaminophen, or a combination of acetaminophen and codeine.

### Outcomes

The primary outcome was CPSP at 12 months after breast cancer surgery. According to the 2016 International Association for the Study of Pain (IASP) criteria (20), CPSP is defined as pain that occurs after surgical intervention and lasts for at least three months, excluding other potential causes (e.g. cancer recurrence and infection). In our study, patients with modified Brief Pain Inventory (mBPI) > 0 at 12 months after breast cancer surgery in surgical area (breast, axilla, and arm) were considered to develop CPSP (21).

Outcomes of breast cancer were also recorded within 12 months, including 1) recurrence of breast cancer in the ipsilateral breast, thoracic wall, and axillary tissue with pathological confirmation; 2) distant metastasis, including the occurrence of breast cancer in the contralateral breast or any other remote organs with pathological confirmation, or multiple lesions consistent with metastases found on imaging examination; and 3) death from any reasons.

### Data acquisition

Baseline characteristics including demographics, medical insurance level, preoperative data, surgical data, pathology data, and adjuvant therapies after surgery were acquired from the dataset of the RCT (NCT00418457). In addition, pain-related data including the presence of persistent pain of any kind, preoperative pain in the operative area (breast, axilla, and arm), opioid (fentanyl) consumption during surgery, postoperative pain intensity ratings within 2, 24, and 48 hours after surgery were also recorded in the dataset. Postoperative pain intensity was categorized according to verbal numerical rating scale (NRS) from no pain (NRS 0), mild pain (NRS 1~3), to moderate-to-severe pain (NRS 4~10).

Outcome observation and follow-up information were also obtained from the dataset of the RCT. All follow-ups were done using one qualified investigator unaware of the patient’s random assignments and intraoperative management, and they have tried to contact not only the patients, but also their families and caregivers, and tried at least 3 times at each follow-up time point (30 days, 3 months, 6 months, and 12 months after surgery) to ensure a high proportion of successful follow-up (> 99%). Patients who withdrew from the RCT, lost to follow-up, or had missing data were excluded from our study.

### Statistical analysis

The full dataset was randomly split into a training dataset (90%) and a testing dataset (10%). Feature selection and model-development were performed in the training dataset, and the validation and evaluation of the models were performed in the testing dataset. Continuous variables were converted to restricted cubic splines for better fitting (22).

Recursive feature elimination (RFE) (23) is a feature selection method that fits a model and removes the weakest features until the specified number of features is reached. Features are ranked by the model’s coefficient or feature importance attributes and attempted to eliminate dependencies and collinearity that may exist in the model by recursively eliminating a small number of features per loop. In this study we used RFE for variables selection and pre-set 5 as the minimum number of variables. And then two clinical experts identified the final variables incorporated into the prediction model based on the results of RFE, clinical experience and risk factors mentioned in most studies (11, 12, 24, 25). In addition, we analyzed the contribution (gain) of each identified variable to 12-month CPSP.

In the model-development phase, four prediction models including conventional logistic regression, RF, GBDT and XGBoost algorithm models, were constructed with the identified variables for CPSP prediction, the overview of these four models are shown in **Table 1**. Ten folds Grid-search cross-validation (26) was used to select the best tuning parameters for the RF, GBDT, and XGBoost models. The performances of these four models were validated and evaluated using the area under the receiver operating characteristic curve (AU-ROC) for discrimination and the integrated calibration index (ICI), E50, E90 and Hosmer-Lemeshow test for calibration (27). The diagnostic accuracy, including sensitivity, specificity, positive and negative likelihood ratios (PLR and NLR), and positive and negative predictive values (PPV and NPV), was also calculated to compare the clinical validity of different models.

**Table 1:** Overview of four prediction models.

Prediction models	Overview
Logistic regression	An extension of the linear regression model for classification problems, used to examine the association of categorical or continuous independent variable(s) with one binary outcome, more suitable for linear data pattern.
RF	An ensemble machine learning method for both classification and regression tasks, training a large number of individual decision trees on various sub-samples of the dataset, and using average to improve the predictive accuracy and control over-fitting, more suitable for nonlinear data pattern.
GBDT	An iterative decision tree algorithm with strong generalization ability, which combines several weak prediction models (predictors with poor accuracy) into a strong learner (a model with strong accuracy), performed well in both classification and regression tasks but with over-fitting.
XGBoost	Built on the GBDT framework and designed for supervised learning tasks such as regression, classification and ranking, which is a more regularized model formalization to control over-fitting.

GBDT, gradient boosting decision tree; RF, random forest; XGBoost, extreme gradient boosting.

All statistical analyses were performed by R 4.0.2 and python 3.8.0. A 2-sided *P-*value less than 0.05 was considered the threshold for statistical significance.

## Results

### Baseline characteristics

**Figure 1:**
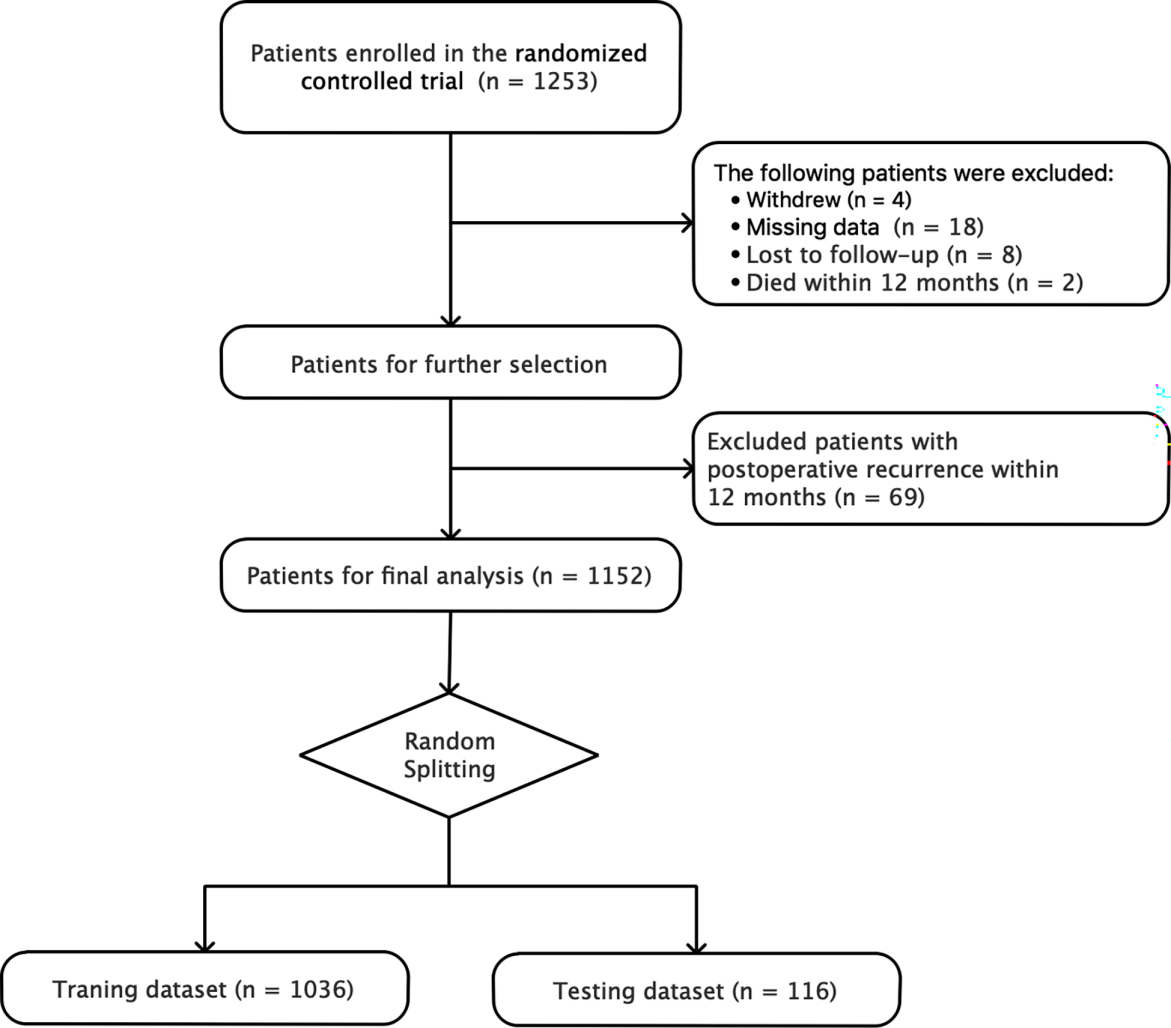
Flow chart of patient selection and random splitting.

A total of 1152 patients were finally included in the study, excluding 8 lost to follow-up, 4 withdrew from the RCT, 18 having missing data, 2 died within 12 months, and 69 with postoperative recurrence within 12 months. The process of patient selection is shown in **Figure 1**. 255 (22.1%) patients developed CPSP at 12 months after breast cancer surgery. According to the results of univariable analyses in the full dataset using logistic regression, in those patients with 12-month CPSP, the age, medical insurance level, menstruation status, whether receiving axillary lymph node dissection (ALND), surgical technique, and acute postoperative pain (within 2 days after surgery) differed significantly from those in patients without CPSP. Data is shown in **Table 2**.

**Table 2:** Baseline characteristics and univariable analyses for chronic postsurgical pain (CPSP) in full dataset.

Variables	CPSP (n = 255)	No CPSP (n = 897)	Unadjusted OR (95% CI)	*P* value
**Preoperative factors**				
Medical insurance level				
No	113 (44.3)	473 (52.7)	Reference	
NCMS	17 (6.7)	94 (10.5)	0.76 (0.43 to 1.32)	0.326
UEBMI+URMI+CMI	125 (49.0)	330 (36.8)	1.59 (1.19 to 2.12)	0.002
Age, y	50 ± 9	49 ± 10	1.02 (1.00 to 1.03)	0.016
BMI, kg/m^2^	23.7 ± 3.3	23.8 ± 3.2	0.99 (0.95 to 1.04)	0.805
Smoking history	4 (1.6)	11 (1.2)	1.28 (0.41 to 4.07)	0.671
Alcohol use	1 (0.4)	3 (0.3)	1.17 (0.12 to 11.33)	0.890
ASA physical status				
1	173 (67.8)	643 (71.7)	Reference	
2~3	82 (32.2)	254 (28.3)	1.20 (0.89 to 1.62)	0.234
Menstruation status				
Pre-menopausal	98 (38.4)	414 (46.2)	Reference	
Peri-menopausal	23 (9.0)	113 (12.6)	0.86 (0.52 to 1.42)	0.554
Post-menopausal	134 (52.5)	370 (41.2)	1.53 (1.14 to 2.06)	0.005
Depression	1 (0.4)	6 (0.7)	0.58 (0.07 to 4.88)	0.620
Chronic ß-blocker use	4 (1.6)	22 (2.5)	0.63 (0.22 to 1.86)	0.406
Preoperative pain in the operative area	126 (49.4)	439 (48.9)	1.02 (0.77 to 1.35)	0.894
History of operation ^*^				
0	107 (42.0)	437 (48.7)	Reference	
1	93 (36.5)	292 (32.6)	1.30 (0.95 to 1.78)	0.102
≥ 2	55 (21.6)	168 (18.7)	1.34 (0.92 to 1.94)	0.125
Preoperative neoadjuvant therapy	8 (3.1)	31 (3.5)	0.90 (0.41 to 1.99)	0.804
Tumor location				
Left	121 (47.5)	462 (51.5)	Reference	
Right	130 (51.0)	424 (47.3)	1.17 (0.88 to 1.55)	0.271
Bilateral	4 (1.6)	11 (1.2)	1.39 (0.43 to 4.44)	0.580
**Intraoperative factors**				
ALND	225 (88.2)	747 (83.3)	1.51 (0.99 to 2.29)	0.056
Surgical technique				
Simple mastectomy	23 (9.0)	134 (14.9)	Reference	
Modified radical mastectomy	180 (70.6)	614 (68.5)	1.71 (1.06 to 2.74)	0.026
Wide local excision with node dissection	40 (15.7)	94 (10.5)	2.48 (1.39 to 4.41)	0.002
Others	12 (4.7)	55 (6.1)	1.27 (0.59 to 2.73)	0.539
Anesthetic technique				
GA	128 (50.2)	443 (49.4)	Reference	
PPA	127 (49.8)	454 (50.6)	0.97 (0.73 to 1.28)	0.820
Pathology stage, tumor (T)				
T0	1 (0.4)	7 (0.8)	Reference	
T1	145 (57.3)	513 (57.6)	1.98 (0.24 to 16.21)	0.525
T2	90 (35.6)	314 (35.2)	2.01 (0.24 to 16.52)	0.517
T3	10 (4.0)	28 (3.1)	2.50 (0.27 to 22.93)	0.418
T4	1 (0.4)	1 (0.1)	7.00 (0.22 to 226.00)	0.272
Tis	6 (2.4)	28 (3.1)	1.50 (0.15 to 14.57)	0.727
Pathology stage, nodes (N)				
N0	145 (57.1)	506 (56.4)	Reference	
N1	52 (20.5)	228 (25.4)	0.80 (0.56 to 1.13)	0.205
N2	28 (11.0)	83 (9.3)	1.18 (0.74 to 1.88)	0.493
N3	29 (11.4)	80 (8.9)	1.27 (0.80 to 2.01)	0.320
Tumor TNM stage				
0	5 (2.0)	32 (3.6)	Reference	
1	95 (37.5)	336 (37.6)	1.81 (0.69 to 4.77)	0.231
2	94 (37.2)	355 (39.8)	1.69 (0.64 to 4.47)	0.286
3	59 (23.3)	170 (19.0)	2.22 (0.83 to 5.97)	0.113
**Postoperative factors within 12 months**				
NRS within 2 days after surgery				
0	7 (2.7)	60 (6.7)	Reference	
1~3	123 (48.2)	547 (61.0)	1.93 (0.86 to 4.32)	0.111
4~10	125 (49.0)	290 (32.3)	3.69 (1.64 to 8.31)	0.002
Postoperative analgesic	61 (23.9)	239 (26.6)	0.87 (0.63 to 1.20)	0.382
Radiotherapy	102 (40.0)	333 (37.1)	1.13 (0.85 to 1.50)	0.403
Chemotherapy	190 (74.5)	678 (75.6)	0.94 (0.69 to 1.30)	0.725
Endocrine treatment	166 (65.1)	608 (67.8)	0.89 (0.66 to 1.19)	0.421

Results presented as x ± s or n (%).

ALND, axillary lymph node dissection; ASA, American society of anesthesiology; BMI, body mass index; CI, confidence interval; CMI, commercial medical insurance; CPSP, chronic postsurgical pain; GA, fentanyl with sevoflurane general anesthesia; NCMS, the new cooperative medical scheme for rural residents; NRS, numerical rating scale; OR, odds ratio; PPA, paravertebral block with propofol general anesthesia; UEBMI, the urban employee basic medical insurance; URMI, the urban resident medical insurance program for self-employed and unemployed urban residents.

^*^ Previous history of operation refers to any type of operation to any body region.

### Features selected in models

**Figure 2:**
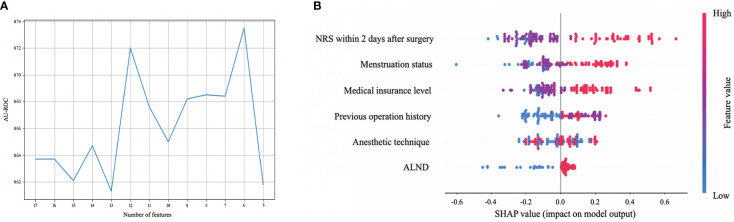
Feature selection for chronic postsurgical pain (CPSP) and the contribution (gain) analysis. **(A)** Feature selection using recursive feature elimination (RFE). The X axis is the number of remaining variables in RFE. Set the minimum number of variables to 5. The Y axis is the area under the receiver operating characteristic curve (AU-ROC) when different variables remain. AU-ROC is the best (0.735) when there are 6 variables left. **(B)** Shapley additive explanation (SHAP) summary plots of the top 6 predictors of the XGBoost algorithm for CPSP. SHAP value reflects the feature importance of every observation. The higher the SHAP value of a feature, the higher the risk of CPSP. A dot is created for each feature attribution value for the model of each patient. Dots are colored according to the values of features for the respective patient and accumulate vertically to depict density. Red represents higher feature values, and blue represents lower feature values. ALND, axillary lymph node dissection; NRS, numerical rating scale.

RFE was used for feature selection. As shown in **Figure 2A**, according to the results of RFE, better discrimination appeared when 12 or 6 variables remained (AUC was 0.720 and 0.735 respectively). Considering the clinical practicability of models, we hoped to select as few predictors as possible when discrimination is similar, so we selected 6 variables to be included, which were medical insurance level, menstruation status, history of any operation to anybody region, anesthetic technique, pathology stage for nodes (stage N), and NRS 2 days after surgery. According to the clinical experience and previous research (3, 5, 25), ALND would be more suitable as one of the selected predictors, replacing the stage N, since ALND may potentially cause nerve damage and is much preferred by breast cancer patients in China for the fear of tumor recurrence, even in patients whose stage N is 0 or 1. In summary, urban medical insurance, including urban employee basic medical insurance (UEBMI), urban resident medical insurance program for self-employed and unemployed urban residents (URMI), and commercial medical insurance (CMI), as well as post-menopausal status, history of at least one operation, under fentanyl with sevoflurane general anesthesia (GA group), receiving ALND and higher NRS within 2 days after surgery were associated with a higher risk of 12-month CPSP after breast cancer surgery.

Furthermore, we analyzed the contribution (gain) of each feature selected above to 12-month CPSP. The importance of each feature listed in the descending order was NRS within 2 days after surgery > menstruation status > medical insurance level > history of operation > anesthetic technique > whether receiving ALND. Details are shown in **Figure 2B**.

### Models development and comparison

We incorporated the 6 predictors mentioned above to construct different prediction models using training dataset, including RF, GBDT and XGBoost algorithm models, and multivariable logistic regression model. The performances of these four models were then evaluated and compared.

**Figure 3:**
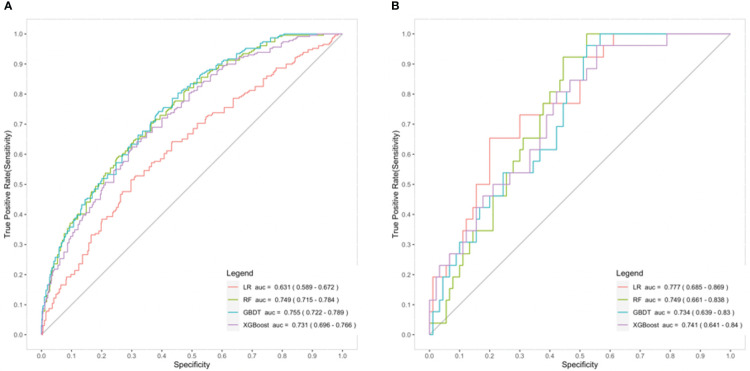
Comparison of the prediction models for chronic postsurgical pain (CPSP) - receiver operating characteristic (ROC) curve in the training dataset **(A)** and the testing dataset **(B)**. AUC, area under the curve; GBDT, gradient boosting decision tree (blue); LR, logistic regression (red); RF, random forest (green); XGBoost, extreme gradient boosting (purple).

The results indicated that machine learning models (RF/GBDT/XGBoost) had better discriminatory power compared with the multivariable logistic regression model [AUC, 0.749 (95% CI 0.715 to 0.784)/0.755 (95% CI 0.722 to 0.789)/0.731 (95% CI 0.696 to 0.766) vs 0.631 (95% CI 0.589 to 0.672)] in the training dataset, while there was no obvious difference among them in the testing dataset [(AUC, 0.749 (95% CI 0.661 to 0.838)/0.734 (95% CI 0.639 to 0.830)/0.741 (95% CI 0.641 to 0.840) vs 0.777 (95% CI 0.685 to 0.870)] (**Figure 3**). As for the calibration evaluated in the testing dataset, the XGBoost model performed better than the multivariable logistic regression model with lower ICI and higher Hosmer-Lemeshow test *P*-value [ICI, 0.050 (95% CI 0.038 to 0.122) vs 0.070 (95% CI 0.050 to 0.146); Hosmer-Lemeshow test *P*-value, 0.829 vs 0.059]. Other machine learning models (RF and GBDT) did not show the apparent superiority in ICI, while they all had higher Hosmer-Lemeshow test *P*-value than logistic regression model [ICI, 0.093 (95% CI 0.053 to 0.168)/0.072 (95% CI 0.049 to 0.156) vs 0.070 (95% CI 0.050 to 0.146); Hosmer-Lemeshow test *P*-value, 0.429/0.384 vs 0.059] (**Table 3**).

**Table 3:** Comparison of prediction models for chronic postsurgical pain (CPSP) in the testing dataset - calibration metrics.

	Logistic regression	RF	GBDT	XGBoost
ICI	0.070 (0.050 to 0.146)	0.093 (0.053 to 0.168)	0.072 (0.049 to 0.156)	0.050 (0.038 to 0.122)
E50	0.070 (0.027 to 0.127)	0.109 (0.034 to 0.170)	0.071 (0.035 to 0.138)	0.046 (0.024 to 0.123)
E90	0.111 (0.095 to 0.307)	0.129 (0.107 to 0.303)	0.114 (0.083 to 0.286)	0.071 (0.064 to 0.257)
H-L *P* value	0.059	0.429	0.384	0.829

Each cell contains the appropriate calibration metric and its 95% confidence interval.

E50 and E90, the median and 90^th^ percentile of the absolute difference between observed and predicted probabilities respectively; GBDT, gradient boosting decision tree; H-L, Hosmer-Lemeshow test; ICI, integrated calibration index (a measure of calibration, which could be interpreted as weighted difference between observed and predicted probabilities); RF, random forest; XGBoost, extreme gradient boosting.

According to the general incidence of CPSP in breast cancer population, we evaluated the clinical validity of all the four prediction models at a 20% risk level of CPSP. As shown in **Table 4**, compared with the multivariable logistic regression model, machine learning models (RF/GBDT/XGBoost) showed higher specificity, PLR and PPV, which could help to identify patients with high risk of CPSP [specificity, 0.914 (95% CI 0.810 to 0.971)/0.922 (95% CI 0.811 to 0.978)/0.907 (95% CI 0.797 to 0.969) vs 0.622 (95% CI 0.514 to 0.722); PLR, 4.200 (95% CI 1.699 to 10.381)/4.315 (95% CI 1.587 to 11.734)/3.658 (95% CI 1.481 to 9.038) vs 2.036 (1.451 to 2.857); PPV, 0.808 (95% CI 0.606 to 0.934)/0.846 (95% CI 0.651 to 0.956)/0.808 (95% CI 0.606 to 0.934) vs 0.370 (95% CI 0.243 to 0.513)], but with lower sensitivity, higher NLR and lower NPV.

**Table 4:** Comparison of prediction models for chronic postsurgical pain (CPSP) in the testing dataset - clinical validity at a 20% risk level.

	Logistic regression	RF	GBDT	XGBoost
Sensitivity	0.769 (0.564 to 0.910)	0.362 (0.240 to 0.499)	0.338 (0.226 to 0.466)	0.339 (0.223 to 0.470)
Specificity	0.622 (0.514 to 0.722)	0.914 (0.810 to 0.971)	0.922 (0.811 to 0.978)	0.907 (0.797 to 0.969)
PLR	2.036 (1.451 to 2.857)	4.200 (1.699 to 10.381)	4.315 (1.587 to 11.734)	3.658 (1.481 to 9.038)
NLR	0.371 (0.181 to 0.762)	0.698 (0.566 to 0.861)	0.718 (0.593 to 0.869)	0.729 (0.598 to 0.888)
PPV	0.370 (0.243 to 0.513)	0.808 (0.606 to 0.934)	0.846 (0.651 to 0.956)	0.808 (0.606 to 0.934)
NPV	0.903 (0.801 to 0.964)	0.589 (0.480 to 0.692)	0.522 (0.414 to 0.629)	0.544 (0.436 to 0.650)

Each cell contains the appropriate value and its 95% confidence interval.

GBDT, gradient boosting decision tree; NLR, negative likelihood ratio; NPV, negative predictive value; PLR, positive likelihood ratio; PPV, positive predictive value; RF, random forest; XGBoost, extreme gradient boosting.

## Discussion

Breast cancer is considered to be the most prevalent cancer in women. As survival improves primarily due to earlier detection and improvements in the therapeutic approaches (28), new challenges emerge. Consistent with previous reports, 22.1% patients in our study developed CPSP at 12 months after breast cancer surgery, which could reduce their quality of life (3–5).

At present, more and more prediction models are established to identify the high-risk CPSP patients in advance (9–12). Sipilä R et al. created a 6-factor risk index to predict persistent pain at 6 months after surgery using Bayesian model in Finland population prospectively (9). Another 4-item preoperative risk score for persistent pain at 4 months after surgery was developed with multivariable logistic regression model in Switzerland (10). Meretoja TJ et al. created a web-based risk calculator using logistic regression analyses to assess the risk of persistent pain at 1 year after surgery in European breast cancer cohorts (11). However, most of these models are conducted in European patients, with a lack of studies on Asian patients, and the low clinical utility limits their application in clinical practice. Machine learning is a form of artificial intelligence that uses computer algorithms to identify nonlinear data patterns within large datasets to formulate outcome prediction (13), which may be more suitable for the prediction for CPSP. Considering the poor performance of machine learning algorithms chosen in previous studies, this time we selected three representative machine learning methods with good fitting ability, including RF, GBDT, and XGBoost to develop prediction models, based on a high-quality dataset and rigorous model evaluation, intending to improve their clinical utility.

Our results suggested that, although the superiority of machine learning models over logistic regression model in discrimination and calibration are not particularly prominent, they performed better in specificity, PLR, and PPV. We found that posterior positive probability, reflected by PPV, was around 0.8 using machine learning methods, which reflects a large increase from the prior probability of 0.2 (22.1%). That means the probability of developing CPSP is as high as 80% if the patient was categorized as high risk using our prediction model. This indeed could improve the models’ clinical utility and help clinicians identify high-risk CPSP patients more accurately and confidently, so that appropriate treatments could be taken promptly. Considering the high quality and low lost to follow-up rate (<1%) of our dataset, these results were reliable and convincing.

According to the feature selection, acute postoperative pain which reflected by a higher NRS within 2 days after surgery, is one of the risk factors for CPSP, consistent with previous research (29). It has been reported that the paravertebral block could improve early postoperative analgesia and prevent CPSP by impacting the transition from acute to chronic pain (7, 8, 30). This may explain why the patients under paravertebral block with propofol general anesthesia (PPA group) had a lower risk of CPSP, compared with those who under fentanyl with sevoflurane general anesthesia (GA group).

Menstruation status was another important predictor for CPSP after breast cancer surgery. It was found in our results that patients under post-menopausal stage seemed to experience a higher risk of CPSP. The decline of estrogen and various musculoskeletal and climacteric symptoms may be the possible explanations (31). However, some reports suggested a younger age was associated with greater risk of CPSP (25). This might be related to the social background difference in different ethnic groups. Post-menopausal women are generally over 55 years old and tend to be retired in China. In most cases, their children, now adults, have left home for work or study, making them experience low hormone levels and the loneliness due to unaccompanied simultaneously. These might lead to a more anxious mental status which could cause or aggravate the feel of pain (32).

Recently Wang Y, et al. also constructed prediction models of chronic pain after breast cancer surgery using a variety of machine learning techniques (33). However, these models not only have lower discriminatory power, sensitivity and PPV, but also do not include psychosocial factors. Pain is a strongly subjective feeling that is affected by the disease itself and relevant clinical factors, as well as social factors, cultural backgrounds, psychological state, and so on. Therefore, in this study, we also took medical insurance level into consideration to represent patients’ social backgrounds and reflect their psychosocial status. According to the current forms of medical insurance in China, we divided patients’ medical insurance level into no medical insurance, rural medical insurance, which is the new cooperative medical scheme (NCMS) for rural residents, and urban medical insurance, which includes UEBMI, URMI and CMI (34). Our results demonstrated that patients with urban medical insurance suffered a higher risk of CPSP. Urban medical insurance could represent the urban residents. They usually have higher economic and educational level and pay more attention about their physical health, life quality, psychological condition and self-feeling, therefore more prone to exacerbate the feeling of pain. Conversely, patients living in rural areas, often with NCMS, may suffer from a heavier burden of life, have a lower level of education and self-focus, and thus seldom care about inconspicuous pain.

Our study also had several limitations. First, it was a secondary analysis based on the dataset in one single center of a multicenter RCT. Although the data in this study were accurate and complete with low drop-out rate, relatively strict inclusion and exclusion criteria in the original trial limited the external validity of our results. Second, despite the machine learning models showed better specificity and PPV, their sensitivity and NPV were not good. Further studies were urgently needed to improve models’ general performance. Third, our prediction model included some specific cultural differences such as insurance in China, which might make the global applicability of the model limited. Finally, psychosocial factors may play an important role in CPSP after breast cancer surgery. Although some psychosocial factors were included in our analyses and model development, they are not enough, especially lacking of data on underlying anxiety. Studies incorporating a more comprehensive analysis of psychosocial factors should be conducted in the future.

## Conclusions

This study developed prediction models for CPSP at 12 months after breast cancer surgery based on machine learning approaches, which could assist clinicians to identify high-risk patients more accurately and conduct clinical interventions in advance. Using machine learning methods could be a novel approach to predict CPSP and help tailoring precise management for patients with breast cancer, leading to a better prognosis and an increased quality of life.

## Data availability statement

The raw data supporting the conclusions of this article will be made available by the authors, without undue reservation.

## Ethics statement

This study was performed in line with the principles of the Declaration of Helsinki. Approval was granted by the Institutional Review Board of our hospital (Number: S-638). The patients/participants provided their written informed consent to participate in the study.

## Author contributions

CS: data acquisition and analysis, manuscript preparation. ML: data acquisition and analysis, tables and figures preparation. LL: data acquisition and analysis, manuscript preparation. LP: study design, data acquisition and analysis, manuscript revision and guarantor. YZ: data analysis and manuscript revision. GT: data acquisition. ZZ: data acquisition. YH: study design and manuscript revision. CS, ML, and LL contributed equally to this study. All authors contributed to the article and approved the submitted version.
